# Cleavage of Kininogen and Subsequent Bradykinin Release by the Complement Component: Mannose-Binding Lectin-Associated Serine Protease (MASP)-1

**DOI:** 10.1371/journal.pone.0020036

**Published:** 2011-05-23

**Authors:** József Dobó, Balázs Major, Katalin A. Kékesi, István Szabó, Márton Megyeri, Krishnan Hajela, Gábor Juhász, Péter Závodszky, Péter Gál

**Affiliations:** 1 Institute of Enzymology, Hungarian Academy of Sciences, Budapest, Hungary; 2 Department of Physiology and Neurobiology, Eötvös Loránd University, Budapest, Hungary; 3 Laboratory of Proteomics, Institute of Biology, Eötvös Loránd University, Budapest, Hungary; 4 School of Life Sciences, Devi Ahilya University, Indore, India; Consejo Superior de Investigaciones Cientificas, Spain

## Abstract

Bradykinin (BK), generated from high-molecular-weight kininogen (HK) is the major mediator of swelling attacks in hereditary angioedema (HAE), a disease associated with C1-inhibitor deficiency. Plasma kallikrein, activated by factor XIIa, is responsible for most of HK cleavage. However other proteases, which activate during episodes of angioedema, might also contribute to BK production. The lectin pathway of the complement system activates after infection and oxidative stress on endothelial cells generating active serine proteases: MASP-1 and MASP-2. Our aim was to study whether activated MASPs are able to digest HK to release BK. Initially we were trying to find potential new substrates of MASP-1 in human plasma by differential gel electrophoresis, and we identified kininogen cleavage products by this proteomic approach. As a control, MASP-2 was included in the study in addition to MASP-1 and kallikrein. The proteolytic cleavage of HK by MASPs was followed by SDS-PAGE, and BK release was detected by HPLC. We showed that MASP-1 was able to cleave HK resulting in BK production. MASP-2 could also cleave HK but could not release BK. The cleavage pattern of MASPs is similar but not strictly identical to that of kallikrein. The catalytic efficiency of HK cleavage by a recombinant version of MASP-1 and MASP-2 was about 4.0×10^2^ and 2.7×10^2^ M^−1^s^−1^, respectively. C1-inhibitor, the major inhibitor of factor XIIa and kallikrein, also prevented the cleavage of HK by MASPs. In all, a new factor XII- and kallikrein-independent mechanism of bradykinin production by MASP-1 was demonstrated, which may contribute to the pro-inflammatory effect of the lectin pathway of complement and to the elevated bradykinin levels in HAE patients.

## Introduction

Bradykinin (BK) is a potent proinflammatory peptide that is released from its precursors high-molecular-weight kininogen (HK) and low-molecular-weight kininogen (LK) by means of limited proteolysis. Plasma kallikrein and tissue kallikrein are the serine proteases that are responsible for BK generation [1], [2]. The release of BK triggers inflammatory reactions via activating endothelial cells resulting in vasodilatation, increased vascular permeability, and production of second-generation mediators such as nitric oxide, prostaglandins and leukotrienes [3]. In patients with hereditary angioedema (HAE) the uncontrolled release of BK leads to recurrent tissue swelling that can be lethal if it occurs in the larynx. HAE can be the consequence of the deficiency of functional C1-inhibitor which is the primary inhibitor of the complement and the contact activation systems [4]–[6].

The contact activation system is the major source of BK production in plasma. It is a proteolytic cascade system in the blood that can be activated by incubation of plasma with negatively charged artificial surfaces (e.g. glass, kaolin) or with certain biological macromolecules (e.g. LPS, amyloid β protein) bound to the surface of different cell types, including endothelial cells, platelets, and polymorphonuclear neutrophils [7]. The first step of contact activation is the autoactivation factor XII. Activated factor XII (factor XIIa) then cleaves and activates factor XI and prekallikrein. Activation of factor XI initiates the intrinsic pathway of coagulation while activation of prekallikrein results in BK production. Most of the prekallikrein (about 85%) can be found in equimolar complex with HK which can bind to cell surfaces [1], [2], [8]. In this way contact system activation results in immediate BK release on the cells, to which its components are bound.

Another major proteolytic cascade system in the blood is the complement system which is an important component of the innate immune system. Activation of the complement system results in the elimination of pathogens and altered self structures (e.g. apoptotic, necrotic cells), and triggers inflammatory reactions [9]. The complement system can be activated through three different activation routes: the classical, the lectin and the alternative pathways [10]. In the case of the lectin pathway pattern recognition molecules (mannose-binding lectin (MBL), ficolins) circulate in the serum that can recognize and bind to different danger signals arisen from invading pathogens or altered self structures. MBL and ficolins form multimolecular complexes with serine proteases (MBL-associated serine proteases = MASPs) that autoactivate upon the recognition molecules bind to the activator structures [11]. One of the MASPs, MASP-2, is able to initiate the complement cascade, since it can cleave C2 and C4, the components of the C3-convertase enzyme complex. MASP-1 however cannot induce C3-convertase formation alone, since it cannot cleave C4. We have shown previously that MASP-1 can exert proinflammatory activities since it can cleave fibrinogen releasing fibrinopeptide A and B [12], and it can stimulate endothelial cells by cleaving the protease-activated receptor 4 (PAR4) [13]. MASP-1 has also been implicated in clot formation both in vitro [14] and in vivo [15].

In the present work we demonstrate that MASP-1 is capable of digesting HK to release BK. This phenomenon can contribute to the initiation of inflammatory reaction during complement activation establishing a stronger innate immune response. MASP-2 can also cleave HK, but cannot release BK. Since C1-inhibitor inhibits the activity of both MASPs [16], [17], it might be possible that the BK-producing activity of MASP-1 can also contribute to the elevated BK level in the case of HAE.

## Materials and Methods

### Materials

Recombinant human MASP-1 and MASP-2 catalytic fragments, rMASP-1 and rMASP-2, were prepared as described previously [16], [18], [19]. These recombinant fragments are composed of the last 3 domains including the catalytic domain, but lack the first 3 domains responsible for the interaction with MBL and ficolins. The concentrations of rMASP-1 and rMASP-2 were calculated using the extinction coefficients ε = 1.54, and 1.88 ml mg^−1^ cm^−1^, and a molecular weights of 45.5, and 43.3 kDa, respectively [18]. Human HK was purchased from Calbiochem (Darmstadt, Germany), or Innovative Research (Novi, MI): both of them were about the same good quality. Human plasma kallikrein (referred to as kallikrein) and human plasma prekallikrein (referred to as prekallikrein) were from Innovative Research (Novi, MI). The molar concentration of kallikrein was calculated based on the product label and a molecular weight of 88 kDa. Human LK was from Sigma (cat. no. K3628 St Louis, MO), however 2 out of 3 batches were already cleaved preventing its use for detailed LK cleavage studies. The selective peptide substrate for MASP-1, LPAPR-AMC, was custom-ordered from Bio Basic (Toronto, Canada). Human C1-inhibitor (Berinert P) was from CSL Behring (King of Prussia, PA). The concentration of C1-inhibitor was calculated using the extinction coefficient ε = 0.382 ml mg^−1^ cm^−1^, and a molecular weight of 71 kDa [20]. All proteins were dissolved in, or buffer-exchanged to HEPES-buffered saline (HBS) buffer (140 mM NaCl, 50 mM HEPES, 0.1 mM EDTA, pH 7.4). BK-acetate was obtained from Sigma (St Louis, MO).

### Differential gel electrophoresis (DIGE)

Human plasma was depleted of 7 abundant components (serum albumin, transferrin, haptoglobin, IgA, IgG, anti-trypsin and fibrinogen) using a Hu-PL-7 multiple affinity removal column (Agilent, Wilmington, DE) according to the instructions of the manufacturer. Total protein concentration in the pooled depleted plasma was determined using the BCA assay (Pierce, Rockford, IL). Depleted plasma at a final plasma protein concentration of 570 µg/ml was incubated in the presence and absence of 10 µg/ml rMASP-1 at 37°C for 1 hour in Tris-buffered saline (TBS) buffer (150 mM NaCl, 20 mM Tris, 0.1 mM EDTA, pH 7.4). Another sample was made with 60 µg/ml C1-inhibitor present in addition to rMASP-1. When the incubation was complete salts and lipids were removed by the method of Santos-González et al. [21]. Finally the dry protein pellets were dissolved in lysis buffer (7 M urea, 2 M thio-urea, 4% CHAPS, 20 mM Tris, 5 mM Mg-acetate, at pH 8.5).

Protocol of sample preparation for DIGE was used as we described previously [22]. Briefly, 50 µg amount of plasma proteins (the untreated, rMASP-1-treated, and the C1-inhibitor + rMASP-1 treated samples, respectively) were labeled with CyDye DIGE Fluor Minimal Labeling Kit (GE Healthcare, Little Chalfont, UK) at a ratio of 400 pmol/50 µg protein according to the instructions. The three differently marked samples were multiplexed and resolved in the same gel. Six gels were prepared. Following electrophoresis, gels were scanned in a Typhoon Trio+ laser scanner (GE Healthcare) using appropriate lasers and filters with the photomultiplier tube biased at 580 V. Images in different channels were overlaid using selected colors and differences were visualized using Image Quant software (GE Healthcare). Differential protein analysis was performed using DeCyder software package 6.0 (GE Healthcare). Spots, the intensity of which appeared or increased on rMASP-1 treatment, and disappeared or decreased on the addition of C1-inhibitor were considered MASP-1 cleavage products.

For protein identification 800 µg of rMASP-1-treated (as above) plasma proteins were run on the 2D gel electrophoresis system as described above, but omitting the labeling step. Gels were stained with Coomassie Brilliant Blue G (Sigma, St Louis, MO), and spots of identified MASP-1 cleavage products were excised from the gel. Protein identification was carried out in the Proteomics Central Facility, University of Szeged (Szeged, Hungary) by MALDI-TOF mass spectrometric analysis of the peptides obtained by tryptic digestion of soaked gel slices [22].

### HPLC and MS

HK (200 µg/ml) was incubated with 0.5 µg/ml kallikrein, 25 µg/ml rMASP-1, 25 µg/ml rMASP-2, or rMASP-1 and rMASP-2 combined (both 25 µg/ml) in HBS (140 mM NaCl, 50 mM HEPES, 0.1 mM EDTA, pH 7.4) buffer in a final volume of 500 µl. After 3 hours at 37°C samples were mixed with 250 µl of 1% TFA 2% acetonitrile to stop the reaction. 500 µl of the samples prepared as above (corresponding to 333 µl of the original mixture) were run on a Source 5RPC ST 4.6/150 (GE Healthcare, Uppsala, Sweden) column, and eluted with a 2–80% acetonitrile gradient in 0.1% TFA. Synthetic BK-acetate served as a control. The amount of BK released was estimated by comparing the area under the curve (AUC) of the BK peak to that of a known amount of pure BK applied. Electrospray ionization mass spectrometric analysis was carried out by directly injecting the presumed BK-containing fractions into a HP-1100 series HPLC-ESI-MS system (Hewlett-Packard, San Diego, CA, USA). The flow rate of 10 mM ammonium-formiate buffer was 0.2 ml/min. The drying nitrogen gas flow rate was 10 l/min at 300°C at a pressure of 30 PSI and the capillary voltage was 4000 V. Total ion current (TIC) chromatogram was obtained in positive ion mode by scanning in the 300–2000 mass/charge range. The mass information was evaluated using Agilent ChemStation software.

### SDS-PAGE, densitometry and regression analysis

HK or LK (200 µg/ml) was incubated with 0.5 µg/ml kallikrein, 25–50 µg/ml rMASP-1, or 25–50 µg/ml rMASP-2 in HBS buffer at 37°C for 0–900 minutes as required. Samples were run on standard 12.5% SDS-PA gels [23] under reducing conditions, except that twice the normal concentration of Tris was used in the separating gel. The heavy and light chains of HK are separated under these conditions. Gels were scanned using the BioRad (Hercules, CA) Gel Doc XR system. Densitometric analysis was done with the Quantity One 4.6.3 software (BioRad, Hercules, CA) in volume analysis mode. A good approximation of the catalytic constants was obtained by fitting the intensity (I) of the intact HK band using the I = I_o_×e^−kobs×t^ equation, where k_obs_ = [E_T_]×k_cat_/K_M_, and [E_T_] is the total enzyme concentration.

### MBL-MASP complex: preparation and HK cleavage

The complexes of MBL and MASPs were prepared essentially by the method described by Tan et al. [24]. Briefly: plasma was thawed, precipitated with PEG3350, then the dissolved pellet was loaded to mannose-sepharose (Sigma, St Louis, MO) in TBS-Ca buffer (150 mM NaCl, 50 mM Tris, pH 7.8, 20 mM CaCl_2_, 0.05% Tween-20), eluted with the same buffer containing 10 mM EDTA instead of CaCl_2_. After adding CaCl_2_ to 40 mM final concentration the partially purified complex was loaded to maltose-sepharose (Sigma, St Louis, MO), and eluted with TBS-Ca containing 100 mM N-acetyl-glucosamine. The activity of MASP-1 in the preparation was determined using the LPAPR-AMC substrate as described [13]. Comparing the activity of plasma MASP-1 to the activity of known concentration of rMASP-1 allowed us to estimate the concentration of MASP-1 in the MBL-MASP preparation. 1.5 µl of 2 mg/ml HK (200 µg/ml final) was added to 13.5 µl aliquots of MBL-MASP complexes and incubated at 37°C for 0–6 hours. The samples were analyzed by reducing SDS-PAGE. Densitometry and regression was done as described in the previous section.

## Results

### MASP-1 and MASP-2 can cleave HK

**Figure 1:**
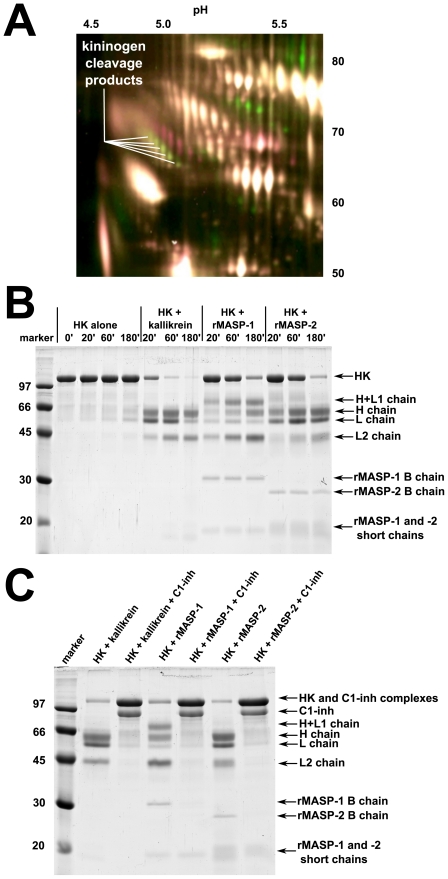
Cleavage of HK by rMASP-1, rMASP-2 and kallikrein, and the effect of C1-inhibitor. (**A**) Kininogen cleavage products identified by 2D-DIGE. Depleted human plasma was incubated alone or in the presence of rMASP-1 in TBS buffer (see Methods). Green spots represent products present in the rMASP-1 treated sample only. Approximate isoelectric points are indicated on the top and molecular weights on the side. (**B**) HK (200 µg/ml) was incubated with kallikrein (0.5 µg/ml), rMASP-1 (25 µg/ml), or rMASP-2 (25 µg/ml) in HBS buffer (see Methods) at 37°C. Samples were taken periodically (time in minutes indicated), and 15 µl aliquots were run on SDS-PA gels under reducing conditions. (**C**) C1-inhibitor (150 µg/ml) prevented cleavage of HK when added to a set of samples prepared as above.

The initial hit that MASP-1 is able to cleave kininogen(s) came from 2D-DIGE analysis of depleted human plasma treated with a recombinant MASP-1 catalytic fragment (rMASP-1) in a search to find novel MASP-1 substrates. Actually a “train” of kininogen cleavage products was the most pronounced difference on the 2D gel of the rMASP-1-treated and the untreated plasma sample (**Figure 1A**). Therefore we undertook to examine the effect of rMASP-1 on isolated HK. As controls plasma kallikrein and a similar recombinant version of MASP-2, named rMASP-2, were included. We found that rMASP-1 is indeed able to cleave HK, although slower than kallikrein. Interestingly rMASP-2 was also able to cleave HK at a somewhat slower, but comparable rate as rMASP-1 (**Figure 1B**).

C1-inhibitor is known to inhibit all 3 proteases [1], [4], [16], [17]. In order to check weather C1-inhibitor is able to prevent cleavage of HK by rMASP-1, rMASP-2 or kallikrein, a molar excess of C1-inhibitor (compared to the protease) was added to HK before the addition of the protease to a set of samples. As expected (**Figure 1C**) degradation of HK was completely prevented in all three cases by the addition of excess C1-inhibitor.

### BK release

**Figure 2:**
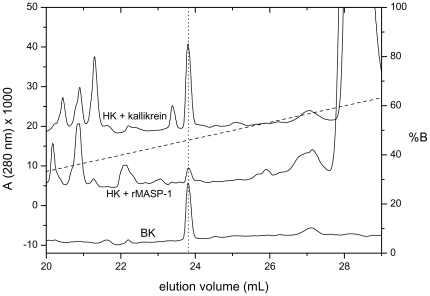
HPLC analysis of HK samples treated with kallikrein or rMASP-1. Samples were run on a Source5 RPC ST 4.6/150 column as described in the Methods. The vertical dotted line indicates the elution position of BK. Dashed line is the percentage of the eluent (%B). Synthetic BK (500 ng) served as a control. BK released by rMASP-1 was confirmed by MS.

The release of BK was monitored by reverse-phase HPLC. The chromatograms on **Figure 2** illustrate the cleavage products obtained by prolonged incubation of HK in the presence of kallikrein, or rMASP-1. In both cases a peak with the same elution time as synthetic BK could be observed. Subsequent MS analysis revealed that the molecular weight of the species in the peak was identical with that of BK. Consequently cleavage of HK by rMASP-1 results in BK release, similar to kallikrein.

It should be noted that according to our calculations MASP-1 produced approximately 25–30% of the potentially releasable BK from HK, whereas kallikrein released about 90–100% during the time course of the experiment.

In the case of rMASP-2 we could not detect a BK peak by HPLC (data not shown), therefore it cannot produce detectable amounts of BK from HK on its own in spite of the fact that it cleaves HK seemingly very much alike kallikrein (**Figure 1B**). It is likely that the BK segment remains attached to the H (or the L) chain upon HK cleavage by MASP-2.

**Figure 3:**
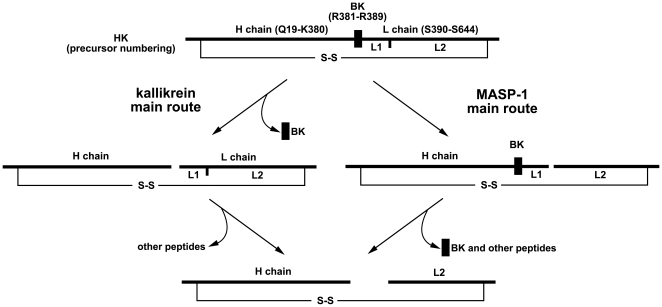
Simplified fragmentation profile of HK produced by the action of kallikrein and rMASP-1. Only the fragments that explain the patterns observed on SDS-PAGE are indicated, however it should be noted that the release of BK requires two cleavage steps, which are combined on this figure.

All proteases can cleave HK at other positions as well, since besides the BK peak, peaks of other unidentified peptides are present in the chromatograms (**Figure 2**). A likely source of these peptides is the L1 segment (**Figure 3**) of the light (L) chain, since the disulfide bonded heavy (H) and L2 chains are known to form a stable end product [25], [26] in the case of kallikrein-treated HK, and the same can be observed in the case of rMASP-1 and rMASP-2, too.

### Cleavage pattern of HK

**Figure 4:**
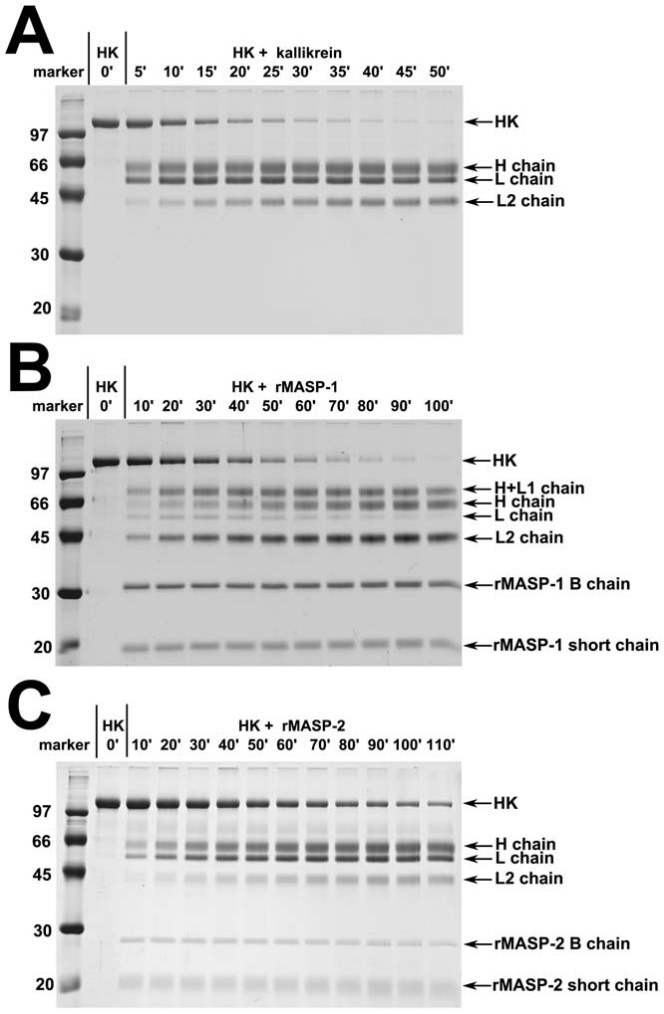
Kinetics of HK cleavage. HK (200 µg/ml) was incubated with (**A**) kallikrein (0.5 µg/ml), (**B**) rMASP-1 (50 µg/ml), or (**C**) rMASP-2 (30 µg/ml) in HBS buffer (see Methods) at 37°C. Samples were taken periodically (time in minutes indicated), and 15 µl aliquots were run on SDS-PA gels under reducing conditions. The kinetic constants, determined by densitometry of the HK band followed by non-linear regression analysis, are found in Table 1.

As it can be observed on **Figure 1B** (and also on **Figure 4**) the cleavage pattern of HK by rMASP-1 is somewhat different from that of by kallikrein, whereas rMASP-2 digests HK in a way seemingly similar to kallikrein. **Figure 3** depicts the likely cleavage pattern of kallikrein and MASP-1. In the case of kallikrein, HK is converted to a heavy (H) and a light (L) chain first [1], [26] releasing BK form the H chain shortly afterwards. Since BK release requires two cleavage steps its production is somewhat delayed [27] compared to the initial cleavage of HK. A third slower cleavage occurs within the L chain between R437 and K438 [26] producing the end product of HK degradation. It seems like that in the case of rMASP-1 the fastest cleavage occurs at or near this site because only a small amount of L chain is produced, and a fragment larger that the H chain appears on the reduced SDS-PA gels. In the case of rMASP-2 the cleavage pattern is similar to the one by kallikrein (**Figure 3**), except that BK remains attached to the H (or L) chain.

### Kinetics of HK cleavage

HK was incubated in the presence of kallikrein, rMASP-1, or rMASP-2 at 37°C, samples were taken periodically, the reaction was stopped by adding reducing SDS-PAGE sample buffer, and the samples were analyzed by SDS-PAGE (**Figure 4**). The kinetic constants (k_cat_/K_M_) were determined by densitometric analysis of the intact HK bands followed by non-linear regression analysis. rMASP-1 cleaved HK at a rate about 300–400-fold slower, whereas rMASP-2 cleaved it about 500–600-fold slower than kallikrein (**Table 1**). These values represent the overall initial (i.e. at any site) cleavage rates and BK release is somewhat delayed compared to these values as explained before. In plasma MASP-1 and MASP-2 are found associated with MBL and ficolins, and their activation is linked [28], hence in plasma the effect of the two enzymes might be additive in term of HK cleavage. We tested the possibility that MASP-2 can also influence the BK production. We found however that the presence of active MASP-2 did not change the amount of BK released by MASP-1 (data not shown).

**Table 1:** The observed rate constants (k_obs_) and the calculated catalytic efficiencies (k_cat_/K_M_) of HK cleavage by kallikrein and MASPs.

protease	enzyme conc. [E_T_]	k_obs_ (s^−1^)	k_cat_/K_M_ (M^−1^ s^−1^)	relative activity
kallikrein	5.7×10^−9^ M	8.6±0.8×10^−4^	1.5±0.1×10^5^	1
rMASP-1	1.1×10^−6^ M	4. 5±0.2×10^−4^	4.0±0.2×10^2^	1/375
rMASP-2	0.7×10^−6^ M	1.9±0.1×10^−4^	2.7±0.2×10^2^	1/555

### LK cleavage by rMASP-1

**Figure 5:**
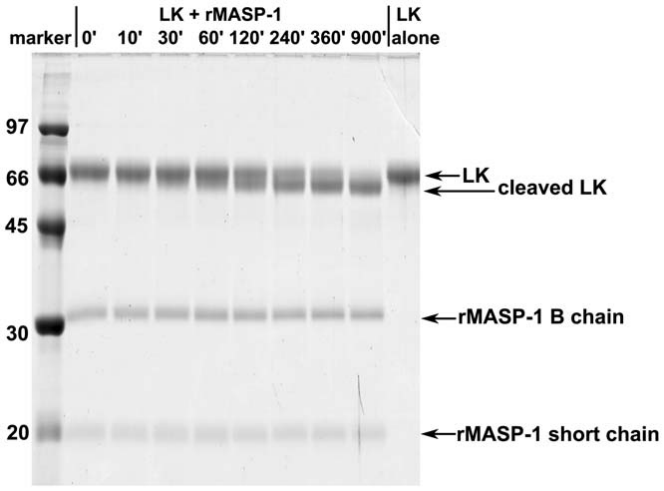
LK cleavage by rMASP-1. LK (200 µg/ml) was incubated in HBS buffer (see Methods) at 37°C (time in minutes indicated) in the presence of 50 µg/ml rMASP-1. 10 µl samples per lane were analyzed by reducing SDS-PAGE. Densitometric analysis revealed that the cleavage rate of rMASP-1 on LK is about 5 times lower than that on HK.

Our preliminary experiments show that LK is also cleaved by rMASP-1 (**Figure 5**). The cleavage rate of LK is about 5-times lower (k_cat_/K_M_∼80 M^−1^s^−1^) than that of HK, hence this process is likely to be less relevant. Kallikrein can also cleave LK, however this process is thought to be less significant physiologically [1], [29] than HK cleavage.

### rMASP-1 does not activate plasma prekallikrein

**Figure 6:**
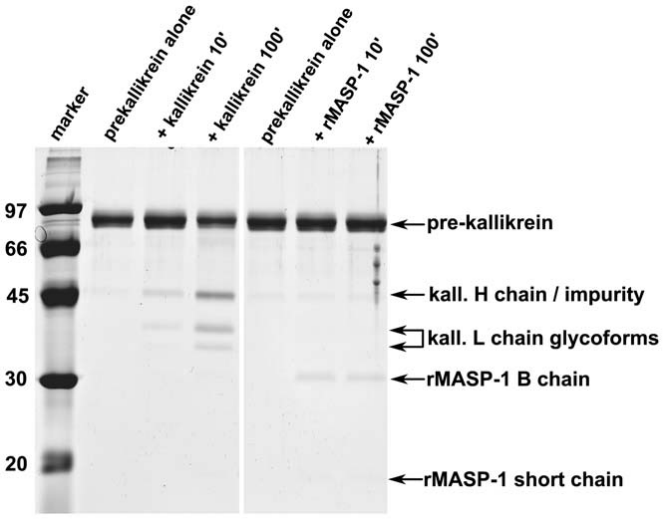
rMASP-1 does not activate prekallikrein. Prekallikrein (100 µg/ml) was incubated in HBS buffer (see Methods) at 37°C (time in minutes indicated) in the presence of 5 µg/ml kallikrein or rMASP-1. 10 µl samples per lane were analyzed by reducing SDS-PAGE. The contrast was somewhat enhanced for the better visibility of faint bands.

The HK preparations used can potentially contain contaminating proteases. To test for this possibility HK was incubated in the reaction buffer, and it was not significantly degraded in 3 hours at 37°C (**Figure 1B**). Another possibility is that HK may be contaminated by a small amount of prekallikrein, and if rMASP-1 is able to activate prekallikrein, then cleavage of HK would be due to the kallikrein formed by the action of rMASP-1 and not to rMASP-1 itself. In order to exclude this possibility we examined the action of rMASP-1 on prekallikrein. As shown on **Figure 6** prekallikrein is not activated by rMASP-1 during prolonged incubation at 37°C. rMASP-2 also did not cause any significant prekallikrein activation (data not shown), whereas kallikrein resulted in slow activation of prekallikrein as expected [30] (**Figure 6**). It was reported earlier that Heat Shock Protein 90 catalyzes the activation of prekallikrein but only if prekallikrein is bound to HK [31]. We tested whether MASPs are able to activate prekallikrein in the presence of excess HK, however we could not detect any activation of prekallikrein (data not shown).

### The complexes of MBL and MASPs also cleave HK

**Figure 7:**
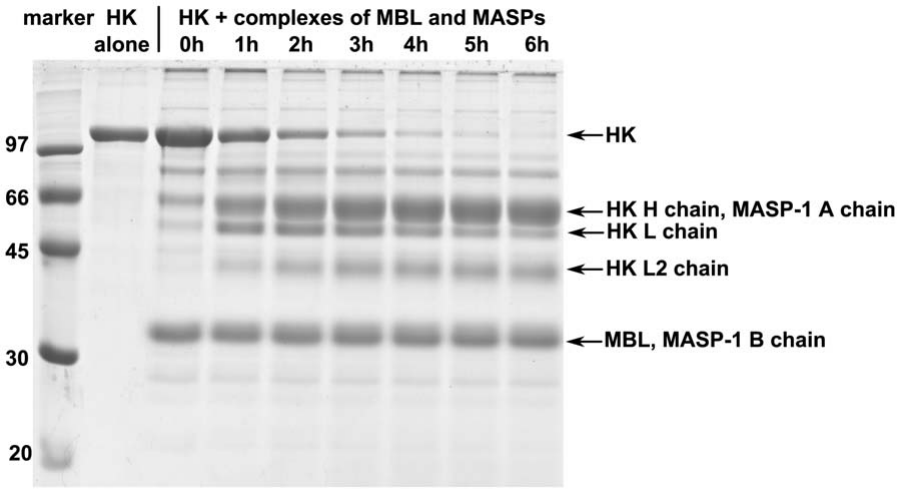
Cleavage of HK by MBL-MASP complexes. HK (200 µg/ml final) was added to the MBL-MASP complexes isolated from human plasma in TBS-Ca buffer (see Methods) and incubated at 37°C (time in hours indicated). The MASP-1 concentration was approximately 60 nM (∼5 µg/ml). 15 µl samples per lane were analyzed by reducing SDS-PAGE.

Physiologically MASP-1 and MASP-2 are in complexes with MBL and ficolins. We prepared partially purified MBL-MASPs complexes. Adding HK to the MBL-MASPs complexes resulted in cleavage of HK in a similar fashion as that of by rMASP-1 and rMASP-2. The cleavage occurs in the presence and absence of Ca^2+^ with similar efficiency (data not shown). The isolated complexes presented in **Figure 7** contained about 60–100 nM MASP-1 determined from the activity measured on a selective MASP-1 substrate [13]. The estimated k_cat_/K_M_ is in the range of 1000–3000 M^−1^s^−1^, which is significantly higher than the value determined for rMASP-1. The cleavage pattern suggests that MASP-2 may also play a role. The MASP-2 concentration in the preparation was not determined, although it is expected to be much less than that of MASP-1 because of its significantly lower serum concentration (MASP-2 = 0.5 µg/ml vs. MASP-1 = 6 µg/ml) [32], [33]. Further studies would be necessary to measure the accurate cleavage rates on separate MBL-MASP-1 and MBL-MASP-2 complexes, and on isolated plasma MASP-1 and MASP-2 however, the preparation of these is not well established.

## Discussion

The lectin pathway of the complement serves as a first line of defense against invading pathogens and it also initiates of clearance of altered host cells. MBL and ficolins (H-, L- and M ficolin) are soluble pattern recognition receptors that form multimolecular complexes with MASPs. MASPs are present as zymogens until the recognition molecules bind to the target structure. Upon such binding the zymogen serine proteases autoactivate and initiate the effector phase of the complement cascade [11]. Up to now three MASPs are known: MASP-1, MASP-2 and MASP-3. The physiological role of MASP-2 is well-known: it can initiate the complement cascade by cleaving C2 and C4 complement components. The role of MASP-3 is still elusive. MASP-1 is an active serum protease and several potential physiological substrates have been proposed [11]–[13], [16], [18]. MASP-1 can cleave complement component C2, but cannot cleave C4 efficiently, therefore it is unable to initiate the complement cascade alone. It was shown however, that MASP-1 can augment the activity of MASP-2 either by activating zymogen MASP-2 or facilitating C3-convertase formation due to its C2 digesting activity [28], [34], [35]. We also showed that MASP-1 can cleave some typical thrombin substrates such as fibrinogen, factor XIII and PAR4 [12], [13]. The crystal structure of MASP-1 [16] suggests that it has more relaxed substrate specificity than related complement proteases having only one or two natural substrates (e. g. C1r, C1s, MASP-2).

In order to identify further potential physiological substrates for MASP-1 in plasma we used a proteomic approach. We incubated human plasma with active recombinant MASP-1 and analyzed the results by differential gel electrophoresis (DIGE). One of the most clear-cut hits was a set of kininogen cleavage products. This result prompted us to analyze the digestion of HK by MASP-1 in detail. We demonstrated that both MASP-1 and MASP-2 are able to cleave HK analogously to plasma kallikrein. HK degradation by MASP-1 also produced BK, while MASP-2 did not release BK. Cleavage of a kallikrein substrate by MASP-1 is supported by the structural data. Overlaying the structures [16], [36], the substrate binding groove of MASP-1 (PDB code 3GOV) seems to be compatible with that of kallikrein (PDB code 2ANY), but not the other way around, i.e. the substrates of kallikrein can probably fit into MASP-1, but the some substrates of MASP-1 probably collide with residue Lys575 (precursor numbering) of kallikrein (data not shown).

In our work we discovered a novel factor XII- and kallikrein-independent mechanism of BK formation. MASP-1 and MASP-2 can be activated during infection or stressful conditions. It was demonstrated previously that MBL can bind to endothelial cells following periods of oxidative stress and initiate the complement cascade [37]. It means that activation of MASPs and MASP-1-mediated HK cleavage can take place on the surface of the endothelial cells where the local concentration of MASPs can be much higher than in serum. This phenomenon can be an important factor of the proinflammatory activity of the complement system which is essential to maintain the homeostasis. Although MASP-1 cannot initiate the complement cascade alone, its activity on HK, fibrinogen and PAR4 can help to mount an even more powerful immune response.

The major source of BK production in serum is the contact activation cascade. Factor XII can autoactivate and subsequently activate prekallikrein to kallikrein by limited proteolysis. Active plasma kallikrein then digests HK liberating the vasoactive peptide BK and it also activates additional zymogen factor XII molecules providing an amplification loop in the kinin cascade. Recently it was demonstrated that prekallikrein also cleaves HK slowly in a stoichiometric fashion [7]. Both factor XIIa and plasma kallikrein can be inhibited by the serpin C1-inhibitor [1]. C1-inhibitor is the major inhibitor of MASP-2 and it also efficiently inhibits MASP-1 [16], [17], [38], [39]. In the case of HAE when C1-inhibitor is absent or dysfunctional, and the complement cascade is increasingly activated [4], [40], [41], uncontrolled cleavage of HK by MASP-1 may contribute to the elevated BK level. Since MASP-1 autoactivates spontaneously in the absence of inhibitors, it is possible that this protease cleaves HK, even in the absence of any contact system activator, contributing to the elevated baseline level of BK observed in HAE patients [7], [42]. On the other hand special activation of MASP-1 occurring during infection, oxidative stress or possibly other conditions may initiate HAE attacks. It is plausible that under these conditions the MASP-1-mediated BK release may contribute to the worsening of the symptoms, however further experiments are needed to clarify the exact role of MASP-1 in HAE.
